# Diminished Vitamin D Receptor Protein Levels in Crohn’s Disease Fibroblasts: Effects of Vitamin D

**DOI:** 10.3390/nu12040973

**Published:** 2020-04-01

**Authors:** Laura Gisbert-Ferrándiz, Jesús Cosín-Roger, Carlos Hernández, Dulce C. Macias-Ceja, Dolores Ortiz-Masiá, Pedro Salvador, Juan V. Esplugues, Joaquín Hinojosa, Francisco Navarro, Sara Calatayud, María D. Barrachina

**Affiliations:** 1 Departamento de Farmacología and CIBER, Facultad de Medicina, Universidad de Valencia, 46010 Valencia, Spain; laura.gisbert@uv.es (L.G.-F.); pedro.salvador@uv.es (P.S.); juan.v.esplugues@uv.es (J.V.E.); sara.calatayud@uv.es (S.C.); 2 Fundación para la Investigación Sanitaria y Biomédica de la Comunitat Valenciana, FISABIO, 46015 Valencia, Spain; jesus.cosin@uv.es (J.C.-R.); carlos.hernandez-saez@uv.es (C.H.); yuche_dulce@hotmail.com (D.C.M.-C.); 3 Departamento de Medicina, Facultad de Medicina, Universidad de Valencia, 46010 Valencia, Spain; m.dolores.ortiz@uv.es; 4 Hospital de Manises, 46940 Valencia, Spain; jhinojosad@gmail.com (J.H.); fran.navarro.vicente@gmail.com (F.N.)

**Keywords:** Crohn’s disease, vitamin D, vitamin D receptor (VDR), fibroblasts, fibrosis

## Abstract

Vitamin D (VD) deficiency has been associated to Crohn’s disease (CD) pathogenesis, and the exogenous administration of VD improves the course of the disease, but the mechanistic basis of these observations remains unknown. Vitamin D receptor (VDR) mediates most of the biological functions of this hormone, and we aim to analyze here the expression of VDR in intestinal tissue, epithelial cells, and fibroblasts from CD patients. The effects of VD on a fibroblast wound healing assay and murine intestinal fibrosis are also analyzed. Our data show diminished VDR protein levels in surgical resections and epithelial cells from CD patients. In intestinal fibroblasts isolated from damaged tissue of CD patients, we detected enhanced migration and decreased VDR expression compared with both fibroblasts from non-damaged tissue of the same CD patient or control fibroblasts. Treatment with VD increased VDR protein levels, avoided the accelerated migration in CD fibroblasts, and prevented murine intestinal fibrosis induced by the heterotopic transplant model. In conclusion, our study demonstrates diminished VDR protein levels associated with enhanced migration in intestinal fibroblasts from damaged tissue of CD patients. In these cells, VD accumulates VDR and normalizes migration, which supports that CD patients would benefit from the VD anti-fibrotic therapeutic value that we demonstrate in a murine experimental model.

## 1. Introduction

Crohn’s disease (CD) is a chronic inflammatory disorder of the gastrointestinal tract characterized by transmural inflammation, which often leads to intestinal fibrosis and the formation of strictures. Current pharmacological anti-inflammatory treatment does not prevent fibrosis in susceptible patients, and surgery is required in a high percentage of patients which, however, does not rule out recurrence [1,2]. In recent years, a better knowledge of the fibrotic pathways has emerged from other organs [3,4], and the assessment of some anti-fibrotic therapies has been proposed for CD patients [5,6]. However, the lack of current clinical trials forces us to better understand the etiopathogenesis of intestinal fibrosis which is finally mediated by the activation/dysregulation of subepithelial myofibroblasts that triggers excessive extracellular matrix (ECM) and collagen deposition [7,8]. 

Clinical studies report serum Vitamin D (VD) levels lower than 20 ng/mL or 50 nmol/L in CD patients, a situation that has been qualified as VD deficiency and explained by the reduced food intake or malnutrition characteristic of these patients [9,10,11]. VD plays an immunomodulatory role in the gut [11], and the exogenous administration of VD to CD patients prolongs periods of clinical remission and decreases the risk of surgery or hospitalization [12,13]. In addition, several studies report that VD improves symptom-based activity scores [14] and the therapeutic response to specific immunosuppressive therapy [15,16,17]. However, little is known about the mechanistic basis that explicates both how VD deficiency contributes to the pathogenesis of CD and the beneficial effects of VD in these patients.

Vitamin D receptor (VDR) is a nuclear transcription factor that mediates most of the biological functions induced by VD [18]. In cultured cells, VD inhibits the ubiquitin-proteasome degradation and increases VDR protein levels [19] which mediates the effects of VD in the gut. We aim to analyze here the basal and VD-stimulated expression of VDR in intestinal fibroblasts isolated from CD patients and the effects of VD in fibroblasts migration and murine intestinal fibrosis.

## 2. Materials and Methods 

### 2.1. Patients

Control and CD patients were recruited from the Surgical Service of the Hospital of Manises (Valencia, Spain), following the Helsinki declaration recommendations. The study was approved by the Institutional Review Board of the hospital (2018/00438/PI). Written informed consent was obtained from all patients. All patients included in the study are Caucasian, and we collected demographic and clinical data from them, including age, sex, and disease behavior (Table 1).

Intestinal resections from control patients, from the stricture in CD patients presenting a stenotic behavior (B2), or from the damaged area of CD patients with a penetrating behavior (B3), were obtained after surgery. In general, in CD patients, the last pharmacological treatment dose was administered at least 3 weeks before surgery. As control samples, we used the non-damaged tissue obtained from patients undergoing surgery due to a colorectal carcinoma.

### 2.2. Primary Fibroblasts and Epithelial Cells Isolation

Primary fibroblasts and epithelial cells were isolated from human intestinal resections of control and CD patients, as previously reported [20,21]. The tissue was cut in small pieces and incubated in agitation with HBSS-EDTA 30 min at 37 °C. After this step, the supernatant was collected and centrifuged to obtain the epithelial cells. Then a digestion of the pieces with collagenase I (1 mg/mL), hyaluronidase (2 mg/mL), and DNAse (1 µL/mL) in PBS was performed during 30 min at 37 °C. Finally, the explants were seeded in a Petri dish with the culture medium. The medium (DMEM high glucose, Sigma-Aldrich) was supplemented with FCS 20%, Penicilin/Streptomicin (100 µg/mL), Gentamycin (100 µg/mL), Amphotericin B (2 µg/mL), and Ciprofloxacin (16 µg/mL). Intestinal fibroblasts from passages 6 to 8 were used in all experiments, and they were treated with 1α,25-Dihydroxyvitamin D_3_ (VD) (D1530; Sigma-Aldrich, Madrid, Spain) 10 nM or 100 nM dissolved in ethanol or vehicle for 24 h. 

### 2.3. Fibroblasts Wound Healing Assay

Standardized wounds in the fibroblast monolayer were made by a single scraping with a disposable pipette tip, as previously reported [22], and medium with or without inactivated fetal bovine serum (iFBS) containing calcitriol 100 nM (D1530, Sigma) or vehicle was added. Then fibroblast photos were taken at different time points. In all cases, the wounded area was determined (ImageJ; National Institutes of Health, Bethesda, MD, USA) from 3 representative photographs taken of each well at 0, 24, and 48 h. Results were expressed as the percentage of the wound at each time point for the maximal wounded area (time 0, 100%). These experiments were performed using an Olympus IX81 (Hamburg, Germany) fluorescence inverted microscope, and the Cell^R software v.2.8 was employed to take images manually.

### 2.4. Immunohistochemical Studies

Immunohistochemistry for VDR was performed in fixed and paraffin-embedded sections (5 µM) of intestinal resections from damaged mucosa of CD patients or healthy mucosa from colorectal cancer patients [23]. 

The heat-mediated antigen retrieval was performed with 10 mM of sodium citrate buffer at pH 6.0 (Dako Target Retrieval Solution) during 20 min at 98 °C. After the inactivation of endogenous peroxidase and blocking the slides during 1 h at room temperature, intestinal tissues were incubated with the primary antibody Anti-VDR antibody (1:200; 12550, Cell Signaling, Danvers, MA, USA) overnight at 4 °C. The samples were incubated for 30 min at room temperature with the Biotinylated Universal Antibody (1:100; BA-1400, Vector, Peterborough, UK) as a secondary antibody. A negative control without the primary antibody incubation was also performed. Afterwards, in order to get a stronger signal, a 30 min room temperature incubation with Vectastain^®^ Universal Elite ABC Kit (Vector) was performed. A signal was developed after 2 min with DAB enhanced substrate (Sigma-Aldrich, Madrid, Spain). All samples were counterstained with hematoxylin, and pictures were obtained with a microscope (Leica DMI 3000).

### 2.5. Mice

Female C57BL/6 mice were used in all experiments (10–12 weeks old, 20–25 g weight). Animals were housed in stainless steel cages in a room kept at 22 ± 1 °C with a 12 h light/12 h dark cycle and had free access to food and water. Care conditions were adapted to facilitate access of the animals to food and water ad libitum during the experiments. All experiments were performed in compliance with the European Animal Research Law, and the protocols were approved by the institutional animal care and use committees of the University of Valencia (2019/VSC/PEA/0290). 

### 2.6. Induction of Intestinal Fibrosis by Heterotopic Transplant of Colonic Tissue and Vitamin D Treatment

The in vivo model of intestinal fibrosis was induced in C57BL/6 mice using a heterotopic intestinal transplant as previously described [21,24]. In this protocol, small pieces of colon were subcutaneously transplanted into the dorsal neck region of recipient mice. After 7 days, recipient mice were sacrificed by neck dislocation, and intestinal grafts were obtained. An adjacent segment of the colon from each donor was kept to be used as an autologous control tissue (named as day 0). 

Recipient mice received intraperitoneally a daily dose of VD or its vehicle until sacrifice. VD (D1530; Sigma-Aldrich) dissolved in ethanol was administered at the dose of 2 μg/kg in a 0.9% NaCl solution.

### 2.7. Sirius Red Staining

Sirius Red staining was performed in intestinal grafts in order to determine the collagen layer in paraffin-embedded tissues (5 µm) as previously described [21] and the staining was examined under transmission light. The collagen layer thickness was quantified in intestinal grafts by using the software Image J.

### 2.8. RNA Extraction and Real-Time Quantitative PCR (RT-qPCR)

Total RNA from fibroblasts and epithelial cells was isolated with Illustra RNAspin Mini RNA isolation Kit (GE Healthcare Life Science), and total RNA from the colonic tissue was obtained using Tripure Isolation reagent (Roche Diagnostics). In both cases, 1 µg was used to obtain cDNA with the PrimeScript RT reagent Kit (Takara Bio Inc.). Real-time PCR was performed with the SYBR^®^ Premix Ex Taq (Takara Bio Inc.) in a LightCycler thermocycler (Roche Diagnostics). Specific oligonucleotides were designed according to the reported sequences and are shown in Table 2 and Table 3. The ΔΔC_T_ method was used to calculate the fold induction of studied genes. β-actin was used as a housekeeping gene.

### 2.9. Protein Extraction and Western Blot Analysis

Homogenization with lysis buffer for cells (50 mM TrisHCl pH 7.8, 137 mM NaCl, 1 mM EDTA, 10 mM NaF, 10 mM β-glycerophosphate, 1 mM Na_3_VO_4_, 1% Triton X-100, 0.2% N-Lauroylsarcosine, and 10% Glycerol) and for colonic tissue (10 mM HEPES pH 7.5, 2 mM MgCl_2_, 1 mM EDTA, 1 mM EGTA, 10 mM NaCl, 10 mM NaF, 0.1 mM Na_3_VO_4_, 1 mM DTT, 10% NP-40, 1 mM PMSF) containing both proteases inhibitors (Complete Mini tablets, Roche Diagnostics) was used to obtain protein lysates. Equal amounts of protein were loaded onto SDS-PAGE gels and analyzed by Western blot, by using specific primary antibodies shown in Table 4. Protein bands were detected with SuperSignal™ West Femto Substrate (ThermoFisher) in a LAS-3000 (Fujifilm). The Image Gauge version 4.0 software (Fujifilm) was used to quantify the protein expression by means of densitometry. Total protein data were normalized to Glyceraldehyde 3-phosphate dehydrogenase (GAPDH).

### 2.10. Statistical Analysis

Data were expressed as mean ± s.e.m. and compared by a *t*-test for comparisons between two groups, and by one-way analysis of variance (ANOVA) with Newman–Keuls post hoc correction for multiple comparisons. A *p* value < 0.05 was considered to be statistically significant. The correlation between different data obtained in human samples was analyzed using Spearman’s correlation coefficient. 

## 3. Results

### 3.1. VDR Expression Is Diminished in Intestinal Resections of CD Patients

**Figure 1:**
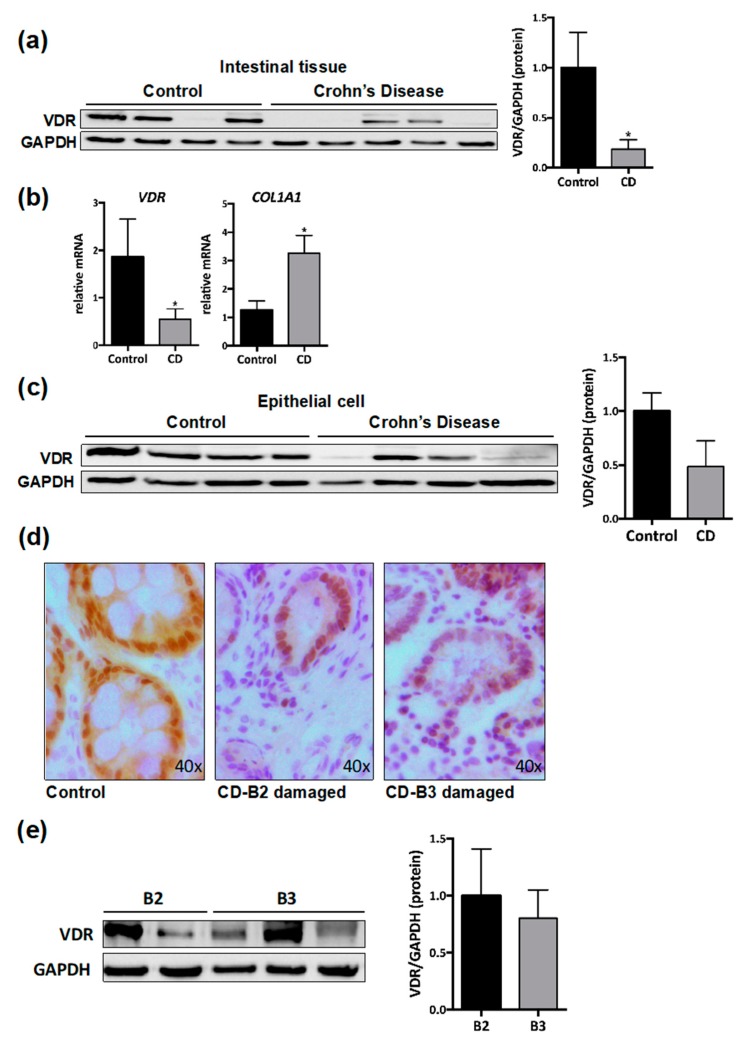
Diminished vitamin D receptor (VDR) expression in damaged intestinal resections from Crohn’s disease (CD) patients. (**a**) A representative Western blot image of VDR protein in lysates of total mucosa from control (*n* = 8) and from CD patients (*n* = 10). The graph shows VDR protein expression vs. Glyceraldehyde 3-phosphate dehydrogenase (GAPDH) represented as fold induction vs. control mucosa. (**b**) mRNA expression (expressed as fold induction vs. control) of different genes vs. β-actin in total mucosa from control (*n* = 5) and CD patients (*n* = 10). In (**a**) and (**b**), bars in graph represent mean ± s.e.m. and significant differences vs. the control group are shown by * *p* < 0.05. (**c**) Representative Western blot from lysates of epithelial cells isolated from intestinal tissue of controls (*n* = 4) and CD patients (*n* = 4). Graph shows protein expression vs. GAPDH represented as fold induction vs. control. (**d**) Representative images showing VDR immunostaining in the mucosa of control and CD patients. (**e**) A representative Western blot image of VDR protein in lysates of total mucosa from CD patients with a stenotic (B2, *n* = 2) or penetrating (B3, *n* = 3) behavior. The graph shows VDR protein expression vs. GAPDH represented as fold induction vs. B2-CD.

In intestinal tissue from CD patients, we detected a diminution of VDR mRNA expression (70.4 ± 32.8%) and VDR protein levels (81.3 ± 32.8%) compared with control tissue (Figure 1a,b). An increase in COL1A1 mRNA expression (139.8 ± 63.26%) was also observed in CD intestine compared with control tissue (Figure 1b). In epithelial crypts isolated from intestinal resections from CD patients, we also found a diminution in protein levels of VDR (51.5 ± 29.2%) compared with those obtain from control tissue (Figure 1c). 

Immunohistochemical analysis in control tissue show cytosolic and nuclear VDR staining in epithelial cells as well as in cells of the lamina propria. In intestinal tissue from CD patients, cytosolic VDR staining was lost, and a slight nuclear VDR staining was detected mainly in epithelial cells (Figure 1d). These changes were detected in samples from CD patients with both a B2 phenotype and a B3 phenotype. VDR protein levels were analyzed by Western blot, and the quantitative analysis reveals non-significant differences among CD behaviors (Figure 1e).

### 3.2. Reduced VDR Protein Levels Are Associated with Increased Migration in Fibroblasts from CD Patients

**Figure 2:**
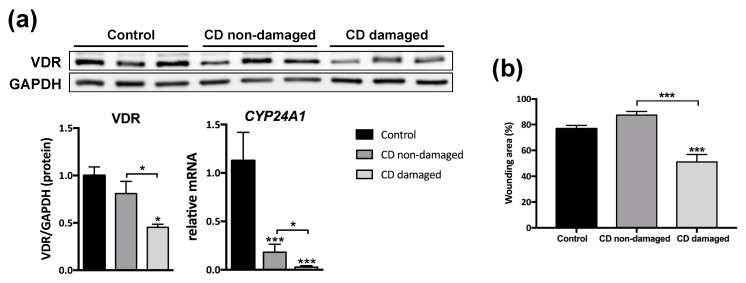
Reduced VDR expression and a higher migration rate in intestinal fibroblasts of CD patients. (**a**) A Western blot showing protein levels in fibroblasts isolated from non-damaged tissue of control patients (*n* = 3) and non-damaged and damaged tissue of CD patients (*n* = 3). Graphs show protein expression vs. GAPDH or the relative mRNA expression of *CYP24A1* gene vs. *β-actin* in control (*n* = 4) and CD (*n* = 7) fibroblasts. In all cases, data are represented as fold induction vs. control fibroblasts. (**b**) The graph represents percentage of the wounding area (time 0, 100%) at 48 h in fibroblasts from control, CD non-damaged and CD-damaged tissue treated with medium iFBS-free. In all cases, bars in graphs represent mean ± s.e.m., and significant differences vs. the control group or vs. the non-damaged CD (connecting lines) are shown by * *p* < 0.05 or *** *p* < 0.001.

Fibroblasts were obtained from intestinal resections of control patients and from damaged and non-damaged intestinal tissue of CD patients. Those obtained from CD-affected mucosa presented significantly lower levels of VDR protein (0.5 ± 0.03) than fibroblasts from both the non-damaged tissue of the same CD patient (0.8 ± 0.1) and control fibroblasts (1 ± 0.1) (Figure 2a). A significant reduction was also detected in the mRNA expression of a VDR target gene, *CYP24A1*, in CD fibroblasts from damaged tissue (0.03 ± 0.013) compared with those from non-damaged tissue (0.18 ± 0.08) and from controls (1.1 ± 0.3) (Figure 2a). Levels of *CYP24A1* were also significantly lower in fibroblast from non-damaged tissue of CD patients than in those from control tissue. Fibroblasts obtained from damaged tissue of CD patients exhibited, 48h after wounding, a lower percentage of wound area (51 ± 5.8) than those obtained from both control (76.8 ± 2.6) or non-damaged tissue of CD patients (87.3 ± 2.88) (Figure 2b). 

### 3.3. VD Increased VDR Protein Levels and Prevented the Accelerated Migration in Fibroblasts from CD Patients

**Figure 3:**
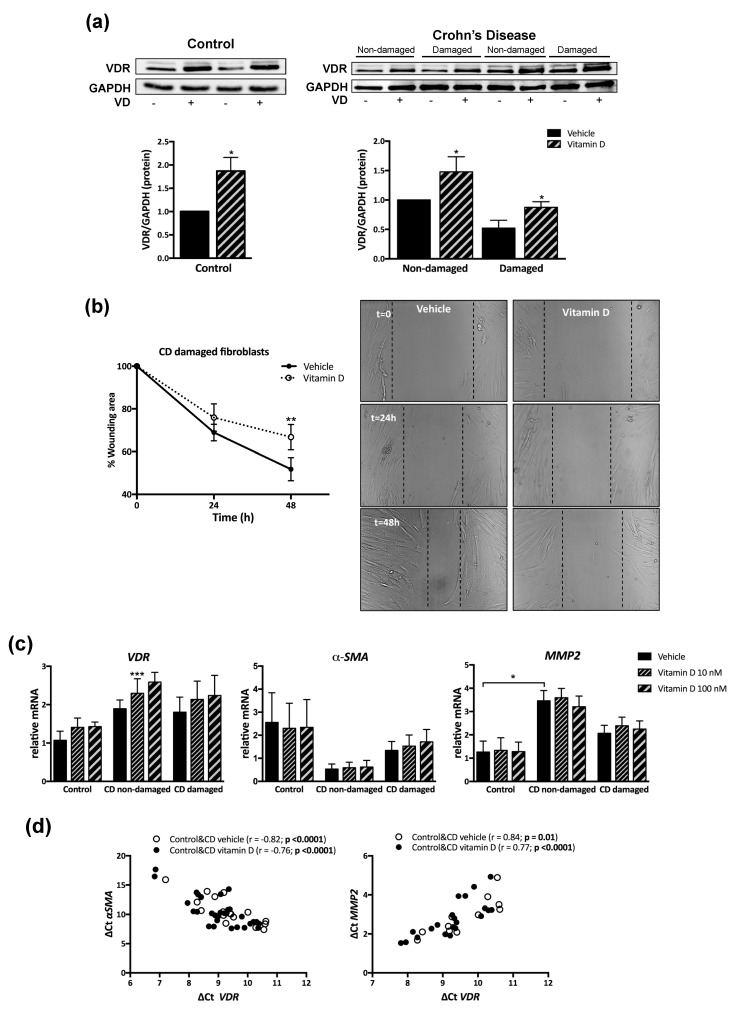
Vitamin D (VD) increased VDR protein levels and prevented enhanced migration in fibroblasts from CD patients. Fibroblasts were treated for 24 h with VD (10 nM or 100 nM) or vehicle. (**a**) A Western blot showing protein levels in fibroblasts isolated from control mucosa (*n* = 4) or the non-damaged and damaged tissue of CD patients (*n* = 4) treated with vehicle or VD (100 nM). Graphs show VDR protein expression vs. GAPDH represented as fold induction vs. vehicle in control cells and vs. non-damaged vehicle in CD cells. Bars represent mean ± s.e.m., and significant differences vs. the respective vehicle group are shown by * *p* < 0.05. (**b**) The graph represents a time course of the percentage of the wounding area (time 0, 100%) in fibroblasts from CD-damaged tissue cultured with medium iFBS-free treated with vehicle (*n* = 4) or VD (100 nM) (*n* = 4). Symbols represent mean ± s.e.m., and significant difference vs. the vehicle group is shown by ** *p* < 0.01. Representative images showing the wound healing assay. (**c**) Graphs show the relative mRNA expression (expressed as fold induction vs. vehicle control group) of different genes vs. *β-actin* in fibroblasts from control mucosa (*n* = 4), CD-non-damaged (*n* = 6), and CD-damaged (*n* = 7) tissue. Bars in graph represent mean ± s.e.m, and significant differences from vehicle-treated control group (connecting lines) are shown by * *p* < 0.05 or from the respective vehicle-treated group by *** *p* < 0.001. (**d**) Significant correlations (showed by Ct gene-Ct *β-actin*) detected between VDR and markers of fibrosis in intestinal fibroblasts treated with vehicle (*n* = 17) or with vitamin D 10 nM and 100 nM (*n* = 34).

VD, compared with vehicle, induced a significant increase in VDR protein levels in control fibroblasts (87 ± 29.2%), in fibroblasts from non-damaged tissue of CD patients (48 ± 25.6%) and in those from damaged intestine (87 ± 31.6%) (Figure 3a). In fibroblasts from damaged tissue of CD patients, VD significantly increased, 48 h after wounding, the percentage of wound (66.75 ± 5.94%) compared to that detected in vehicle-treated cells (51.9 ± 5.4%) (Figure 3b). 

The basal mRNA expression of *VDR*, and α-smooth muscle actin (*α-SMA)* was similar between control fibroblasts (1.1 ± 0.2 and 2.6 ± 1.3, respectively), CD fibroblasts from non-damaged tissue (1.9 ± 0.2 and 0.5 ± 0.2, respectively), and CD fibroblasts from damaged tissue (1.8 ± 0.4 and 1.3 ± 0.4, respectively). Basal metalloproteinase 2 (*MMP2)* mRNA levels were significantly higher in fibroblasts from non-damaged tissue of CD patients (3.5 ± 0.4) than in control cells (1.3 ± 0.5) (Figure 3c). Treatment with VD significantly increase *VDR* mRNA levels, compared with vehicle, in fibroblasts from non-damaged tissue of CD patients (36.2 ± 3.6%) (Figure 3c), while it failed to significantly modify the mRNA expression of *α-SMA* or *MMP2* in any cell analyzed. However, a positive and significant correlation was detected between *VDR* and *MMP2* mRNA expression, while a negative correlation was detected between *α-SMA* mRNA and *VDR* mRNA levels (Figure 3d).

### 3.4. Reduced VDR Expression in Murine Intestinal Fibrosis 

**Figure 4:**
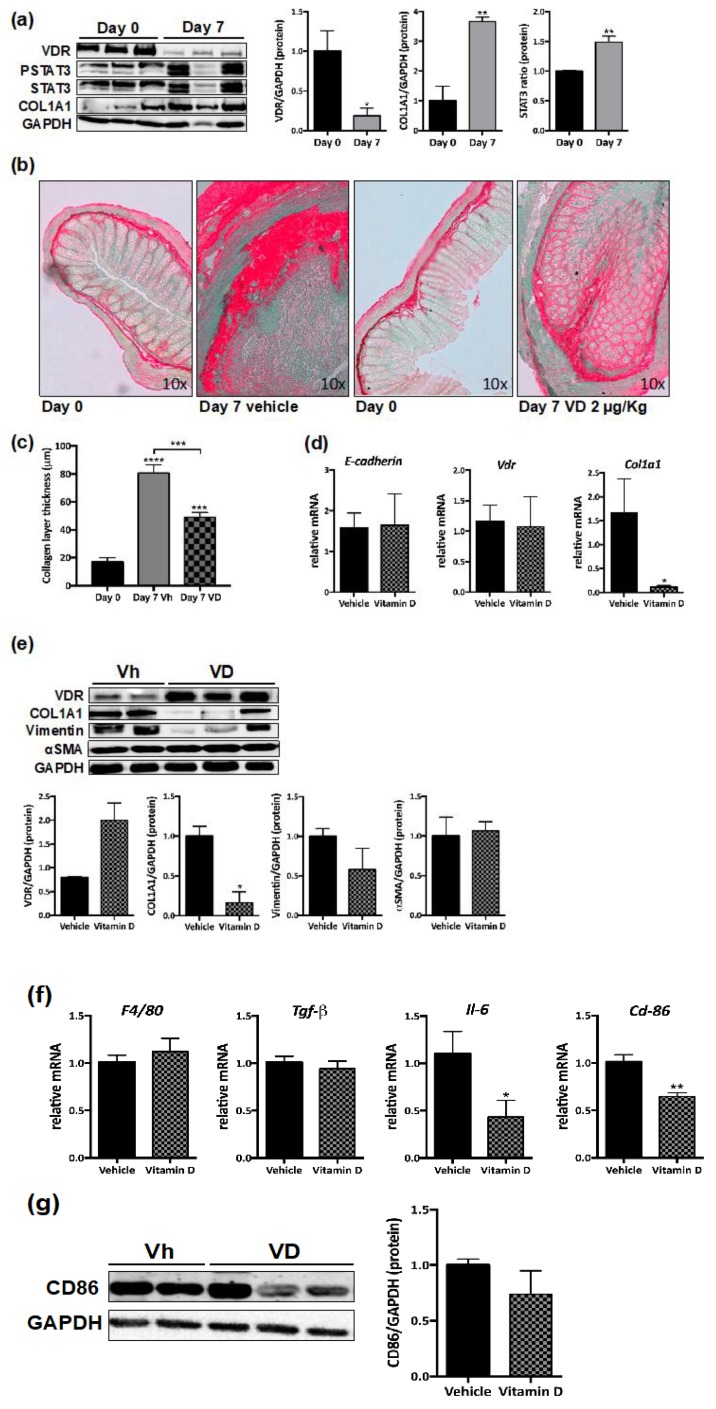
VD reduces murine intestinal fibrosis. (**a**) Western blots of protein levels in total lysates from intestinal grafts at day 0 (control) (*n* = 3) or seven days after transplantation (*n* = 3). Graphs show protein expression vs. GAPDH represented as fold induction vs. day 0. Bars in graph represent mean ± s.e.m., and significant differences vs. day 0 are shown by * *p* < 0.05 or ** *p* < 0.01 (**b**) Sirius Red staining was performed in paraffin-embedded intestinal tissue at day 0 and in intestinal explants. Representative pictures taken under transmission light. (**c**) Graph shows the collagen layer thickness quantified in intestine and grafts by Image J. Significant differences vs. day 0 or vs. 7 days-vehicle (connecting lines) are shown by *** *p* < 0.001. (**d**) Graphs show the relative mRNA expression (expressed as fold induction vs. vehicle-treated group) of different genes vs. β-actin in intestinal explants from mice treated for 7 days with VD 2 μg/kg (*n* = 6) or vehicle (*n* = 6). (**e**) Western blot images of protein expression from 7 day grafts from mice treated with VD (*n* = 3) or vehicle (*n* = 2). Graphs represent protein expression vs. GAPDH quantification expressed as fold induction vs. vehicle-treated group. In (**d**) and (**e**)**,** bars in graph represent mean ± s.e.m. and significant differences vs. the vehicle group are shown by * *p* < 0.05. (**f**) Graphs show the mRNA expression of different genes vs. β-Actin (expressed as fold induction vs. vehicle) in 7 day grafts from mice treated with VD (*n* = 6) or vehicle (*n* = 6). Bars in graph represent mean ± s.e.m., and significant differences vs. the vehicle group are shown by * *p* < 0.05 or ** *p* < 0.01. (**g**) A Western blot showing CD86 protein levels in grafts from vh- or VD-treated mice. The graph represents protein expression vs. GAPDH quantification expressed as fold induction vs. vehicle-treated group.

We next analyzed the protein expression of VDR in the heterotopic transplant mouse model of intestinal fibrosis. As shown in Figure 4a, our results show an important decrease in VDR protein levels (81.6 ± 27.6%) in intestinal grafts obtained 7 days after transplantation in parallel with an increased protein expression of COL1A1 (267.9 ± 51.9%) and the ratio pSTAT3/STAT3 (49 ± 10.1%), compared with colon at day 0.

### 3.5. VD Reduces Murine Intestinal Fibrosis

Next, we proceeded to administer VD (2 μg/kg, i.p.) or its vehicle daily to receptor mice, and grafts were obtained 7 days after transplantation. The histological analysis of the colon shows a decrease in collagen deposition and a preserved histological architecture in colon grafts obtained from VD-treated mice compared with vehicle-treated mice (Figure 4b). A quantitative analysis of the collagen layer thickness reveals higher levels in grafts from vehicle-treated mice (80.8 ± 5.9 µm) compared with both control intestines at day 0 (16.8 ± 3.2 µm) and grafts from VD-treated mice (49 ± 3.4 µm) (Figure 4c). Treatment with VD, compared with vehicle significantly reduced the mRNA expression of *Col1a1* (93.6 ± 38.6%), while it failed to significantly modify the mRNA expression of E-cadherin or *Vdr* at the time point analyzed (Figure 4d). No significant changes were detected in protein expression of VDR in intestinal grafts from VD-treated mice, while a significant reduction in protein levels of COL1A1 (83.2 ± 19.5%) was observed, compared with levels detected in grafts from vehicle-treated mice (Figure 4e). These grafts also exhibited a significant reduction in vimentin protein levels (41.8 ± 35.2%) compared with grafts from vehicle-treated mice, while protein levels of α-SMA were not significantly different (Figure 4e). 

In intestinal grafts from VD-treated mice, the mRNA expression of *F4/80* (1.1 ± 0.1) was not different to that detected in vehicle-treated mice (1.01 ± 0.07) (Figure 4f). However, VD induced a significant decrease (36.4 ± 8.3%) in the mRNA expression of the macrophage marker, cluster of differentiation 86 (*Cd86)* in parallel with a significant diminution in the mRNA expression of interleukin-6 (*Il-6)* (67.1 ± 28.6%) while non-significant differences in *Tgf-β* mRNA expression were observed (Figure 4f). A Western blot analysis reveals a non-significant decrease in CD86 protein levels in grafts from VD-treated mice compared with grafts from vehicle-treated mice (Figure 4g).

## 4. Discussion

VD is at present widely used for the treatment of CD patients because its immunomodulatory effects in the gut, but little is known about its role in intestinal fibrosis, a main feature of CD complications. Our study demonstrates diminished VDR expression in intestinal fibroblasts from CD patients; treatment with VD increases VDR protein levels and prevents the enhanced migration of these cells. In a murine model, the systemic administration of VD reduced intestinal fibrosis. 

The present study shows a significant reduction in VDR protein expression in intestinal resections from CD patients with regard to control tissue, and no differences in VDR expression were observed between the stenotic or penetrating behavior. Immunohistochemical analysis reveals a diminished VDR in both epithelial cells and cells in the lamina propria, and the study performed in isolated crypts reinforces this observation. Previous studies reported diminished VDR levels in intestinal biopsies from patients with inflammatory bowel disease [25], and our results extend these observations by showing for the first time that fibroblasts obtained from the damaged intestine of CD patients present lower VDR protein levels than those obtained from the non-damaged tissue of the same patient. Serum VD levels were correlated with colonic VDR expression in normal mucosa [26], and these levels seem to be dependent on several factors, such as body mass index [27] or sun exposure [28]. However, the difference in VDR protein levels detected in the present study between samples of the same patient, excludes genetics or changes in serum VD levels as responsible for the VDR down-regulation observed in cells coming from damaged tissues. Of interest, this reduced VDR protein expression parallels with no changes in *VDR* mRNA expression at the same time point and remains stable over several passages, which strongly suggests the epigenetic regulation of VDR by local inflammation or damage associated to CD [29]. In this line, the overexpression of both miR-125b [30] and miR27b [31], which were shown to be differentially regulated by CD [32], were associated with reduced VDR protein levels. 

Treatment of intestinal fibroblasts with VD induced a significant VDR accumulation in all cells analyzed, although final protein levels were lower in cells obtained from the affected mucosa of CD patients than in cells from non-affected tissue. Of interest, VD failed to significantly modify *VDR* mRNA expression, which reinforces that VD inhibits the proteasomal degradation of VDR [19], a process that may be overactive in fibroblasts from the CD-damaged tissue. However, the possibility that VD down-regulates the expression of miRNAs involved in VDR reduction, as recently reported in lung fibroblasts and cardiac fibrosis [33,34], cannot be ruled out. Of interest, the reduced VDR levels detected in fibroblasts from damaged tissue of CD patients paralleled with an enhanced migration of these cells, and VD significantly prevented the enhanced migration. These observations, together with the significant correlations detected between the mRNA expression of VDR and MMP2 (positive) and α-SMA (negative), strongly support an anti-fibrotic effect of VD in intestinal tissue, previously reported in several other organs [35,36,37], and suggest a direct effect of this hormone on intestinal fibroblasts. 

Finally, we analyzed the relevance of this signaling pathway in intestinal fibrosis development by testing the effects of VD in a murine model. The heterotopic transplant of colonic tissue provokes submucosal and subserosal fibrosis, significant ECM deposition, and important cellular infiltration and thus resembles some of the CD characteristics [21]. In line with the results obtained in human intestinal samples from CD patients, we found reduced VDR expression in murine fibrotic tissue which was not associated with significant differences in cellular composition. Treatment of mice with daily doses of VD preserved the histological architecture of the colon, reduced collagen deposition, and prevented intestinal fibrosis, which reinforces a previous study in a different experimental model [35]. The modulation of the immune response by VD may be involved in its protective effects since intestinal grafts from VD-treated mice showed an altered pattern of macrophage expression characterized by a reduced mRNA expression of the M1 macrophage marker, *Cd86,* in parallel with a diminished mRNA expression of *Il-6*, a known pro-fibrotic cytokine [38]. However, the fact that no significant differences in CD86 protein levels were detected among treatments, together with the direct effects of VD on intestinal fibroblasts shown in the present study, strongly suggests a role of VD acting on fibroblasts in its anti-fibrotic effects. 

## 5. Conclusions

In conclusion, our study demonstrates that VDR is diminished in fibroblasts isolated from damaged tissue of CD patients, and treatment with VD increased VDR levels and prevented the enhanced migration detected in these cells. These results strongly support that CD patients would benefit from the VD anti-fibrotic therapeutic value clearly demonstrated in a murine experimental model.

## Figures and Tables

**Table 1:** Patients characteristics.

	Control	CD
Number of patients	10	12
Age		
17–40 years	3	5
>40 years	7	7
Sex		
Female	4	7
Male	6	5
Behavior		
B2		6
B3		6

**Table 2:** Sequences of human primers used in real-time PCR.

Gene	Sense (5′-3′)	Antisense (5′-3′)
*VDR*	TGGAGACTTTGACCGGAACG	AAGGGGCAGGTGAATAGTGC
*CYP24A1*	ACCAGGGGAAGTGATGAAGC	TCATCCTCCCAAACGTGCTC
*COL1A1*	GGAGCAGACGGGAGTTTCTC	CCGTTCTGTACGCAGGTGAT
*ACTA2 (α-SMA)*	GACCTTTGGCTTGGCTTGTC	AGCTGCTTCACAGGATTCCC
*MMP2*	CATTCCCTGCAAAGAACACA	GTATTTGATGGCATCGCTCA
*ACTB (β-actin)*	GGACTTCGAGCAAGAGATGG	AGCACTGTGTTGGCGTACAG

**Table 3:** Sequences of mouse primers used in real-time PCR.

Gene	Sense (5′-3′)	Antisense (5′-3′)
*Vdr*	ACAAGACCTACGACCCCACCT	AGCCGATGACCTTTTGGATGCT
*E-cadherin*	ACCCAAGCACGTATCAGGG	ACTGCTGGTCAGGATCGTTG
*Col1a1*	CAGGCTGGTGTGATGGGATT	AAACCTCTCTCGCCTCTTGC
*Adgre1 (F4/80)*	CTTCCCAGAATCCAGTCTTTCC	TGACTCACCTTGTGGTCCTAA
*Tgfb*	GCGGACTACTATGCTAAAGAGG	TCAAAAGACAGCCACTCAGG
*Il-6*	GAGTCCTTCAGAGAGATACAGAAAC	TGGTCTTGGTCCTTAGCCAC
*Cd86*	GCACGGACTTGAACAACCAG	CCTTTGTAAATGGGCACGGC
*Actb (β-actin)*	GCCAACCGTGAAAAGATGACC	GAGGCATACAGGGACAGCAC

**Table 4:** Specific antibodies used for Western blot analysis.

Antibody	Supplier	Dilution
VDR	12550, Cell Signaling	1:1000
COL1A1	84336S, Cell Signaling	1:1000
STAT3	ab68153, Abcam	1:1000
Phospho STAT3	ab76315, Abcam	1:1000
Alpha SMA	PA5-16697, ThermoFisher	1:1000
Vimentin	ab92547, Abcam	1:1000
CD86	ab53004, Abcam	1:1000
GAPDH	G9545, Sigma-Aldrich	1:10000
